# Changes in Cardiac Morphology and Function in Individuals With Diabetes Mellitus: The UK Biobank Cardiovascular Magnetic Resonance Substudy

**DOI:** 10.1161/CIRCIMAGING.119.009476

**Published:** 2019-09-11

**Authors:** Magnus T. Jensen, Kenneth Fung, Nay Aung, Mihir M. Sanghvi, Sucharitha Chadalavada, Jose M. Paiva, Mohammed Y. Khanji, Martina C. de Knegt, Elena Lukaschuk, Aaron M. Lee, Ahmet Barutcu, Edd Maclean, Valentina Carapella, Jackie Cooper, Alistair Young, Stefan K. Piechnik, Stefan Neubauer, Steffen E. Petersen

**Affiliations:** 1 William Harvey Research Institute, NIHR Barts Biomedical Research Centre, Queen Mary University of London, United Kingdom (M.T.J., K.F., N.A., M.M.S., S.C., J.M.P., M.Y.K., M.C.d.K., A.M.L., E.M., J.C., S.E.P.).; 2 Barts Heart Centre, St Bartholomew’s Hospital, Barts Health NHS Trust, London, United Kingdom (M.T.J., K.F., N.A., M.M.S., S.C., J.M.P., M.Y.K., M.C.d.K., A.M.L., S.E.P.).; 3 Division of Cardiovascular Medicine, Radcliffe Department of Medicine, University of Oxford, John Radcliffe Hospital, United Kingdom (E.L., A.B., V.C., S.K.P., S.N.).; 4 Department of Cardiology, Copenhagen University Hospital Herlev- Gentofte, Hellerup, Denmark (M.T.J.).; 5 Department of Cardiology, Copenhagen University Hospital Rigshospitalet, Denmark (M.T.J.).; 6 Department of Biomedical Engineering, King’s College London, United Kingdom (A.Y.).

**Keywords:** cardiomyopathies, cardiovascular, diabetes mellitus, heart disease, magnetic resonance imaging

## Abstract

**Background:**

Diabetes mellitus (DM) is associated with increased risk of cardiovascular disease. Detection of early cardiac changes before manifest disease develops is important. We investigated early alterations in cardiac structure and function associated with DM using cardiovascular magnetic resonance imaging.

**Methods:**

Participants from the UK Biobank Cardiovascular Magnetic Resonance Substudy, a community cohort study, without known cardiovascular disease and left ventricular ejection fraction ≥50% were included. Multivariable linear regression models were performed. The investigators were blinded to DM status.

**Results:**

A total of 3984 individuals, 45% men, (mean [SD]) age 61.3 (7.5) years, hereof 143 individuals (3.6%) with DM. There was no difference in left ventricular (LV) ejection fraction (DM versus no DM; coefficient [95% CI]: −0.86% [−1.8 to 0.5]; *P*=0.065), LV mass (−0.13 g/m^2^ [−1.6 to 1.3], *P*=0.86), or right ventricular ejection fraction (−0.23% [−1.2 to 0.8], *P*=0.65). However, both LV and right ventricular volumes were significantly smaller in DM, (LV end-diastolic volume/m^2^: −3.46 mL/m^2^ [−5.8 to −1.2], *P*=0.003, right ventricular end-diastolic volume/m^2^: −4.2 mL/m^2^ [−6.8 to −1.7], *P*=0.001, LV stroke volume/m^2^: −3.0 mL/m^2^ [−4.5 to −1.5], *P*<0.001; right ventricular stroke volume/m^2^: −3.8 mL/m^2^ [−6.5 to −1.1], *P*=0.005), LV mass/volume: 0.026 (0.01 to 0.04) g/mL, *P*=0.006. Both left atrial and right atrial emptying fraction were lower in DM (right atrial emptying fraction: −6.2% [−10.2 to −2.1], *P*=0.003; left atrial emptying fraction:−3.5% [−6.9 to −0.1], *P*=0.043). LV global circumferential strain was impaired in DM (coefficient [95% CI]: 0.38% [0.01 to 0.7], *P*=0.045).

**Conclusions:**

In a low-risk general population without known cardiovascular disease and with preserved LV ejection fraction, DM is associated with early changes in all 4 cardiac chambers. These findings suggest that diabetic cardiomyopathy is not a regional condition of the LV but affects the heart globally.

**Clinical Perspective**

Diabetic cardiomyopathy has typically been described as a condition related to changes in the left ventricle (LV). In the present study, participants from the UK Biobank without known cardiovascular disease and with preserved LV ejection fraction (EF) were examined with cardiac magnetic resonance imaging to study early changes in cardiac morphology and function associated with diabetes mellitus. We find that diabetes mellitus is associated with discrete but significant cardiac remodeling affecting all 4 cardiac chambers. LV volumes were smaller and mass-to-volume ratios were larger in diabetes mellitus despite no differences in LVEF or LV mass. Furthermore, subtle changes in cardiac LV deformation could be detected using cardiac magnetic resonance imaging-tagging even in the presence of preserved EF. Changes in the right ventricle (RV) related to diabetes mellitus has so far remained largely unexplored. In parallel with our findings in the LV, diabetes mellitus was also associated with smaller RV volumes without changes in RVEF. A consistent pattern also emerged for both atria, demonstrating smaller left atrial volumes, smaller right atrial volumes, and lower atrial emptying fractions, which occurred despite no changes in LVEF or RVEF. Thus, our findings suggest that diabetic cardiomyopathy is not a regional condition of the LV but affects the heart globally. These changes can be observed despite no impairment in LVEF or RVEF and before manifest heart disease develops. The present findings therefore significantly add to our current understanding of diabetic cardiac complications and open a new direction for early detection and research into diabetic cardiomyopathy.

## Introduction

**See Editorial by Lima and Xie**

Globally, >500 million people currently have diabetes mellitus (DM) and this prevalence is expected to increase in the coming decades.^1^ Cardiovascular disease (CVD) is the leading cause of death in DM, and the risk of mortality is doubled compared to individuals without DM.^2,3^ Accelerated heart failure is a common manifestation of CVD in patients with DM and can be unrelated to macrovascular ischemic heart disease.^4,5^ A special subset of heart disease in DM has been proposed, diabetic cardiomyopathy, which can lead to diastolic and systolic heart failure.^4,6,7^

The hemodynamic and biomechanical evidence of early changes related to DM stems from echocardiography, suggesting premature diastolic dysfunction,^8,9^ and, in the later stages, affected systolic function. Diabetic cardiomyopathy has been described in 3 stages: the early stage with normal left ventricular (LV) size, mass, and wall thickness, and only discrete changes in diastolic function; the second stage, characterized by abnormal diastolic function and no or only discrete changes in systolic function; and the late stage of diabetic cardiomyopathy where both systolic and diastolic function are affected.^10–12^

Typical early morphological findings relating to DM, as currently understood, are LV hypertrophy and decreased LV chamber size, often with preserved LV ejection fraction (LVEF).^13^ Discrete changes in LV systolic function have been detected using sensitive methods, such as speckle-tracking echocardiography and cardiovascular magnetic resonance imaging (CMR) tagging.^14–17^ Small studies suggest that DM could also potentially affect right ventricular (RV) function. Changes in RV morphology and function related to DM, however, are not well described.^18–20^

CMR is, at present, considered the method of choice for measuring cardiac morphology and function. Compared to echocardiography, CMR demonstrates superior reproducibility, better interobserver and intraobserver variability, better imaging quality, and use of fewer geometric assumptions.^21^ Improved image quality is particularly relevant in patients with DM, where echocardiography can often be difficult to perform because of concomitant obesity. Measurement of strain using CMR-tagging, which at present is the CMR-modality of choice for measuring deformation, can provide insights into discrete myocardial dysfunction.^22^

In the present study within the UK Biobank cohort, we investigated how the presence of DM is associated with cardiac morphology and function in a subsample of participants who has undergone CMR. We hypothesized that CMR would detect early cardiac changes related to DM in a low-risk general population without known CVD and with preserved ejection fraction.

## Methods

### Study Population

The UK Biobank is a large prospective cohort study of ≈500 000 unselected community volunteers aged 40 to 69 at the time of enrollment, living in the United Kingdom. The design and conduct of the study have both been described in detail previously.^23^ The UK Biobank encourages and provides as wide access as possible to its data and samples for health-related research in the public interest by all bona fide researchers from the academic, charity, public, and commercial sectors, both in the UK and internationally, without preferential or exclusive access for any user. Data can be sought directly from UK Biobank via online application at http://www.ukbiobank.ac.uk/register-apply/.

The present study population consisted of the 5065 individuals who underwent CMR examination as part of the pilot phase (April 2014–August 2015) of the UK Biobank imaging enhancement.

**Figure 1.**
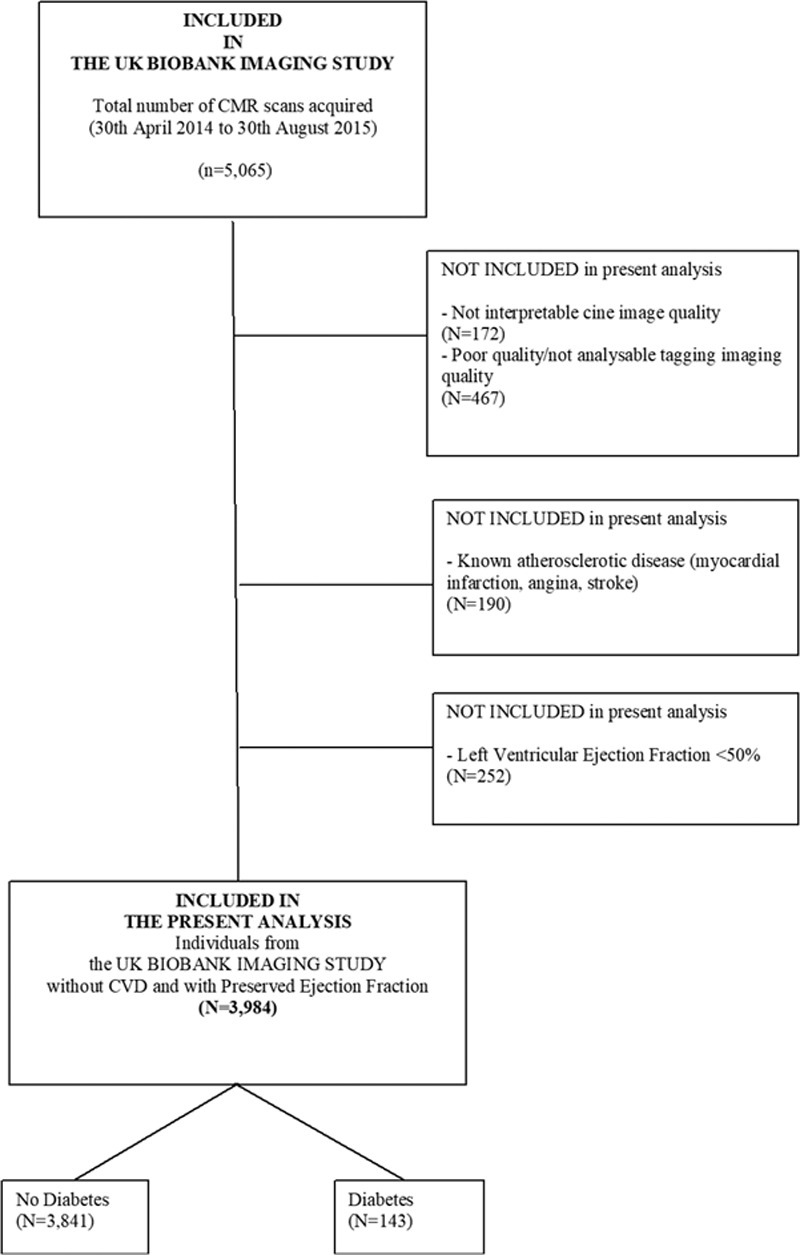
**Flow chart.** CMR indicates cardioac magnetic resonance imaging; and CVD, cardiovascular disease.

In the present study, 172 participants were excluded due to poor cine image quality, 467 were excluded due to poor quality/not analyzable tagging imaging quality, 190 participants were excluded due to known CVD, and 252 participants were excluded due to a LVEF below 50% (Figure 1). Thus, the final population included participants without known CVD and preserved ejection fraction.

### CMR Protocol and Image Analysis

The UK Biobank CMR protocol has been described in detail elsewhere.^24^ In brief, a wide-bore 1.5 Tesla scanner (MAGNETOM Aera, Syngo Platform VD13A, Siemens Healthcare, Erlangen, Germany) was used in all participants. LV and RV long-axis cines and a short-axis stack of balanced steady-state free precession cines were acquired using following typical parameters: Repetition Time/Echo Time=2.6/1.1 ms, flip angle 80°, Grappa factor 2, voxel size 1.8 mm×1.8 mm×8 mm (6 mm for long axis).

Manual analyses were performed of the LV, RV, left atrium (LA), and right atrium (RA) by observers across 2 core laboratories. Analysis software was cvi42 (Version 5.1.1, Circle Cardiovascular Imaging Inc, Calgary, Canada). LV papillary muscles were included in blood pool volumes and thus excluded from LV mass. Detailed methodology and intraobserver and interobserver variability has been described elsewhere.^25^ Investigators were blinded to DM status.

The LV mass/volume ratio was determined by dividing the LV mass by the LV end-diastolic volume. Mass/volume ratio indexes wall thickness to cavity size and is conceptually equivalent to the echocardiogram-derived relative wall thickness (twice the posterior wall thickness divided by the LV end-diastolic diameter).^26^ Atrial emptying fraction was calculated as ([maximum atrial size−minimum atrial size]/maximum atrial size). Atrial and ventricular measures were assessed in absolute measures and also indexed to body surface area using Du Bois formula.^27^

### CMR Tagging

Semiautomated analysis of tagged cine images was performed using CIM software (CIMTag2D v8.1.5 software, Auckland MRI Research Group, New Zealand), which has been validated previously in phantoms and patients.^28^ A grid was aligned automatically to the myocardial tagging planes at end diastole. End systole was determined visually, and tags were manually adjusted at key phases during the cardiac cycle including the end systolic and last frame. Circumferential myocardial strain was calculated by the software from the motion of the intersected tag lines at basal, mid, and apical levels. As previously described, global circumferential strain (GCS) at the mid-level has been shown to have the greatest degree of reproducibility.^29^ Torsion, the wringing motion induced by contracting myofibers in the LV wall during systole, was calculated from the basal and apical strain measures.^30^ For those cases where a basal or apical slice was missing or not analyzable, torsion was calculated between mid-ventricular and the other available slice. Torsion has been shown to be a sensitive marker of myocardial dysfunction.^31,32^

### Participant Characteristics

Comorbidities were determined during the imaging visit by self-reported through an electronic questionnaire and by an interview with a healthcare professional. In cases where data from the imaging visit were unavailable information from the enrollment visit were used except for height, weight, blood pressure, heart rate, and smoking status, which were captured exclusively at the time of imaging. DM status was determined by participants’ response to the binary questionnaire item DM diagnosed by a doctor or self-reported use of DM medication.^33^ Gestational DM alone was determined as no DM. Ethnicity was categorized as white versus nonwhite. Systolic and diastolic blood pressures were defined as the mean of two measurements (Omron 705, OMRON Healthcare Europe, Hoofddorp, the Netherlands). Duration of DM was estimated from self-reported age at DM and age at imaging. HbA1c (glycated hemoglobin) was measured twice in the UK Biobank, first instance was during the initial visit in 2006 to 2010 (n=3752, hereof 135 with DM), second instance was during first repeat visit in 2012 to 2013 (n=1186, hereof 46 with DM); both values are provided in Table 1. Smoking status was defined as a binary variable: current versus nonsmokers at the time of CMR examination. Participants’ level of physical activity was determined by assessing frequency (number of days/wk) and duration (minutes/d) of walking, moderate intensity, and vigorous-intensity exercise. A continuous value for the amount of physical activity, measured in metabolic equivalent minutes/wk, was calculated by weighting different types of activity (walking, moderate, or vigorous) by its energy requirements using values derived from the IPAQ study (International Physical Activity Questionnaire).^34^ The present study population was categorized into tertiles of metabolic equivalent minutes/d and a high physical activity level was determined as the participants in the highest tertile. Use of cholesterol medication, blood pressure medication, and DM medication were determined by self-report. Alcohol consumption was categorized into daily alcohol consumption versus less than daily alcohol consumption by self-report.

**Table 1.** Demographics

	All	No Diabetes Mellitus	Diabetes Mellitus	*P* Value
N	3984	3841	143	
Age, y, mean (SD)	61.3 (7.5)	61.2 (7.5)	63.5 (7.0)	<0.001
Sex, men, N (%)	1792 (45.0%)	1711 (44.5%)	81 (56.6%)	0.004
Ethnicity, nonwhite, N (%)	168 (4.2%)	162 (4.2%)	6 (4.2%)	0.99
BMI, kg/m^2^, mean (SD)	25.8 (4.1)	25.7 (4.1)	28.5 (4.9)	<0.001
Systolic blood pressure, mm Hg, mean (SD)	135.9 (17.9)	135.7 (17.9)	139.7 (16.4)	0.009
Diastolic blood pressure, mm Hg, mean (SD)	78.5 (9.9)	78.5 (9.9)	78.0 (9.7)	0.55
HbA1c, mmol/mol, 2006–2010, median (IQR)	35 (32–37), n=3752	34 (32–37), n=3617	43 (39–52), n=135	<0.001
HbA1c, mmol/mol, 2012–2013, median (IQR)	35 (33–37), n=1186	35 (33–37), n=1140	47 (42–54), n=46	<0.001
Duration of diabetes mellitus, y, median (IQR)	0 (0.0–0.0)	0.0 (0.0–0.0)	7.0 (3.0–15.0)	<0.001
Resting heart rate, beats per minute, mean (SD)	69.6 (11.4)	69.5 (11.3)	72.8 (13.6)	<0.001
Physical activity, MET minutes, highest tertile, N (%)	1348 (33.8%)	1311 (34.1%)	37 (25.9%)	0.040
Current smoker, N (%)	165 (4.1%)	160 (4.2%)	5 (3.5%)	0.69
Daily alcohol, N (%)	726 (18.2%)	701 (18.3%)	25 (17.5%)	0.82
Blood pressure medication, N (%)	749 (18.8%)	672 (17.5%)	77 (53.8%)	<0.001
Cholesterol medication, N (%)	686 (17.2%)	595 (15.5%)	91 (63.6%)	<0.001
Metformin medication, N (%)	77 (1.9%)	NA	77 (53.8%)	NA
Non-metformin medication, N (%)	21 (0.5%)	NA	21 (14.7%)	NA
Insulin medication, N (%)	21 (0.5%)	NA	21 (14.7%)	NA

BMI indicates body mass index; HbA1c, glycated hemoglobin; MET, metabolic equivalent; and NA, not applicable.

### Statistical Analysis

All analyses were performed with STATA 15.1 (STATACorp LP, TX). For demographics, categorical variables were analyzed with the χ^2^ test and continuous variables with Student *t* test.

The association between DM and cardiac measures was analyzed in 3 different linear regression models: a crude model; a model including age and sex; and a multivariable model including age, sex, body mass index, systolic blood pressure, diastolic blood pressure, physical activity (highest tertile versus lowest 2 tertiles), current smoking (yes versus no), daily alcohol consumption (yes versus no), use of blood pressure medication (yes versus no), use of cholesterol medication (yes versus no), and ethnicity (white versus nonwhite). An interaction between DM, RV volumes, and sex has previously been reported.^18^ This, however, was not found in the present study (*P* for interaction >0.9). Other relevant interactions were tested, and none were found to be significant. In a sensitivity analysis, propensity score matching was performed using the covariables from the multivariable model on representative outcomes. A *P*<0.05 was considered statistically significant.

### Ethical Approval

This study was covered by the general ethical approval for UK Biobank studies from the National Health Service National Research Ethics Service on 17 June 2011 (Ref 11/NW/0382). All participants gave written informed consent.

## Results

### Baseline Characteristics

A total of 3984 participants were included, hereof 1792 men (45%), mean age 61.3 years. In the present population, 3.6% of the participants had DM. Participants with DM were more likely to be older, be men, have higher body mass index, higher systolic blood pressure, and be less physically active (Table 1). Also, use of blood pressure and cholesterol medication was more prevalent in the DM population.

In terms of cardiac characteristics, unadjusted measures are shown in Table 2 and suggested differences in LV measures, RV measures, RA measures, and LV strain measures.

**Table 2.** Cardiac Characteristics by CMR

	Value	No Diabetes Mellitus	Diabetes Mellitus	*P* Value
N	3984	3841	143	
Left ventricle				
Left ventricular ejection fraction, %	60.1 (5.4)	60.2 (5.3)	59.7 (5.7)	0.36
Left ventricular mass-to-volume ratio, g/mL	0.6 (0.1)	0.6 (0.1)	0.7 (0.1)	<0.001
Indexed left ventricle				
Left ventricular end-diastolic volume, mL/m^2^	77.0 (13.7)	77.2 (13.7)	72.7 (13.6)	<0.001
Left ventricular end-systolic volume, mL/m^2^	30.8 (7.5)	30.9 (7.5)	29.7 (7.7)	0.093
Left ventricular stroke volume, mL/m^2^	46.2 (8.4)	46.3 (8.4)	43.1 (8.0)	<0.001
Left ventricular mass, mL/m^2^	47.1 (9.7)	47.1 (9.7)	48.3 (9.6)	0.16
Left atrium				
Left atrial emptying fraction, %	66.5 (20.6)	66.4 (20.5)	67.1 (21.6)	0.73
Indexed left atrium				
Left atrial maximal volume, mL/m^2^	36.4 (10.3)	36.4 (10.3)	34.5 (9.2)	0.051
Left atrial minimal volume, mL/m^2^	15.0 (6.6)	15.0 (6.6)	14.8 (6.2)	0.80
Right ventricle				
Right ventricular ejection fraction, %	56.8 (6.2)	56.8 (6.2)	56.9 (6.3)	0.79
Indexed right ventricle				
Right ventricular end-diastolic volume, mL/m^2^	81.8 (15.6)	82.0 (15.5)	76.4 (15.4)	<0.001
Right ventricular end-systolic volume, mL/m^2^	35.6 (9.8)	35.7 (9.8)	33.1 (9.6)	0.004
Right ventricular stroke volume, mL/m^2^	46.2 (8.4)	46.3 (8.4)	43.4 (7.9)	<0.001
Right atrium				
Right atrial emptying fraction, %	78.5 (25.4)	78.7 (25.3)	73.3 (27.5)	0.016
Indexed right atrium				
Right atrial maximal volume, mL/m^2^	43.1 (12.4)	43.2 (12.3)	38.2 (13.6)	<0.001
Right atrial minimal volume, mL/m^2^	24.6 (8.6)	24.7 (8.5)	22.4 (9.8)	0.005
CMR-tagging				
Global circumferential strain, basal, %	17.1 (3.1)	17.1 (3.1)	16.4 (3.6)	0.019
Global circumferential strain, mid, %	19.7 (2.2)	19.7 (2.2)	19.1 (2.3)	<0.001
Global circumferential strain, apical, %	20.8 (3.1)	20.8 (3.1)	20.0 (3.5)	0.003
Torsion, degrees	7.6 (2.0)	7.6 (2.0)	8.0 (2.5)	0.018

Values are displayed as mean (SD). CMR indicates cardiac magnetic resonance imaging.

### LV Morphology and Function—DM Versus No DM

**Figure 2.**
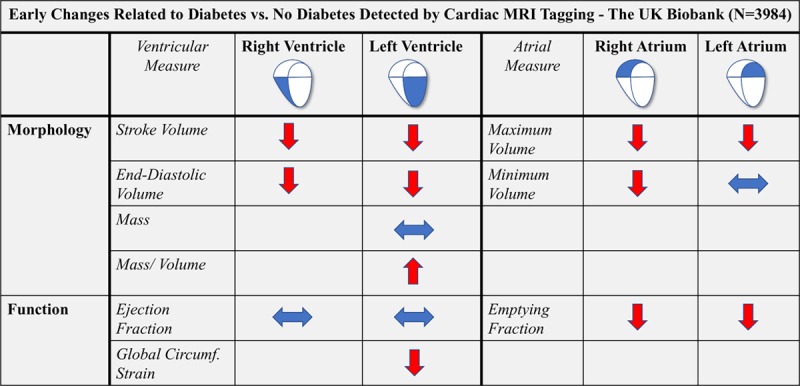
**Principal findings—Early cardiac changes in morphology and function related to diabetes—the UK Biobank Cardiovascular Magnetic Resonance Substudy.** Diabetes mellitus affects all 4 chambers of the heart. While right ventricular ejection fraction (RVEF) and left ventricular ejection fraction (LVEF) are preserved with no difference between diabetes mellitus and no diabetes mellitus, RV and LV chamber sizes are decreased. This occurs before increase in LV mass can be detected but is represented by an increased LV mass-to-volume ratio, suggesting early cardiac remodeling. Deformation imaging with cardiac magnetic resonance imaging (CMR)-tagging shows subtle impairment in LV function related to diabetes despite similar LVEF. The smaller ventricular volumes are accompanied by smaller right atrium (RA) and left atrium (LA) volumes. For both RA and LA, emptying fraction is impaired, which thus represents an early marker of dysfunction occurring before impairments in LV or RV function. Blue arrow: No difference between diabetes mellitus vs no diabetes mellitus. Red arrow UP: Increased in diabetes mellitus vs no diabetes mellitus. Red arrow DOWN: Decreased in diabetes mellitus vs no diabetes mellitus. Empty Field: Not assessed. For values, see Table 2. For coefficients, see Table 3. MRI indicates magnetic resonance imaging.

Table 3 display differences in LV morphology and function in the 3 models: crude; age and sex adjusted; and multivariable adjusted. The principal findings are summarized in Figure 2.

**Table 3.** Difference in Myocardial Morphology and Function using CMR-Tagging in Individuals With Diabetes Mellitus and Without Diabetes Mellitus

	Diabetes Mellitus Versus No Diabetes Mellitus—Crude		Diabetes Mellitus Versus No Diabetes Mellitus—Age and Sex Adjusted		Diabetes Mellitus Versus No Diabetes Mellitus—Multivariable*	
	Coefficient (95% CI)	*P* Value	Coefficient (95% CI)	*P* Value	Coefficient (95% CI)	*P* Value
Left ventricle						
LVEF, %	−0.41 (−1.3 to 0.48)	0.37	−0.30 (−1.2 to 0.6)	0.501	−0.86 (−1.8 to 0.5)	0.065
LV mass/volume, g/mL	0.06 (0.04 to 0.08)	<0.001	0.046 (0.03 to 0.06)	<0.001	0.026 (0.01 to 0.04)	0.006
LVEDV_Indexed_, mL/m^2^	−4.47 (−7.0 to −2.0)	<0.001	−4.84 (−7.2 to −2.5)	<0.001	−3.46 (−5.8 to −1.2)	0.003
LVESV_Indexed_, mL/m^2^	−1.17 (−2.5 to 0.2)	0.09	−1.41 (−2.7 to −0.1)	0.03	−0.42 (−1.7 to 0.9)	0.52
LVSV_Indexed_, mL/m^2^	−3.25 (−4.8 to −1.7)	<0.001	−3.39 (−4.9 to −1.9)	<0.001	−3.00 (−4.5 to −1.5)	<0.001
LVM_Indexed_, g/m^2^	1.26 (−0.5 to 3.0)	0.16	0.26 (−1.2 to 1.7)	0.73	−0.13 (−1.6 to 1.3)	0.86
Left atrium						
LAEF, %	0.61 (−2.9 to 4.1)	0.73	0.32 (−3.1 to 3.7)	0.85	−3.49 (−6.9 to −0.1)	0.043
LAmax_Indexed_, mL/m^2^	−1.91 (−3.8 to 0.0)	0.051	−1.61 (−3.5 to 0.6)	0.098	−2.52 (−4.4 to −0.6)	0.010
LAmin_Indexed_, mL/m^2^	−0.16 (−1.4 to 1.1)	0.80	−0.20 (−1.4 to 1.0)	0.76	−1.01 (−2.3 to 0.2)	0.11
Right ventricle						
RVEF, %	0.14 (−0.9 to 1.2)	0.79	0.40 (−0.6 to 1.4)	0.43	−0.23 (−1.2 to 0.8)	0.65
RVEDV_Indexed_, mL/m^2^	−5.52 (−8.4 to −2.7)	<0.001	−6.23 (−8.8 to −3.7)	<0.001	−4.22 (−6.8 to −1.7)	0.001
RVESV_Indexed_, mL/m^2^	−2.60 (−4.4 to −0.8)	0.004	−3.08 (−4.7 to −1.49)	<0.001	−1.56 (−3.2 to 0.06)	0.059
RVSV_Indexed_, mL/m^2^	−2.90 (−4.4 to −1.4)	<0.001	−3.12 (−4.6 to −1.7)	<0.001	−2.64 (−4.1 to −1.2)	<0.001
Right atrium						
RAEF, %	−5.36 (−9.7 to −1.0)	0.016	−8.18 (−12.1 to −4.2)	<0.001	−6.17 (−10.2 to −2.1)	0.003
RAmax_Indexed_, mL/m^2^	−5.06 (−7.4 to −2.7)	<0.001	−5.87 (−8.1 to −3.6)	<0.001	−3.44 (−5.68 to −1.2)	0.003
RAmin_Indexed_, mL/m^2^	−2.30 (−3.9 to −0.7)	0.005	−3.09 (−4.6 to −1.6)	<0.001	−1.97 (−3.5 to −0.4)	0.012
Left ventricular strain imaging by CMR-tagging						
GCS, basal, %	−0.75 (−1.4 to 0.1)	0.019	−0.60 (−1.22 to 0.02)	0.057	−0.53 (−1.17 to 0.11)	0.10
GCS, mid %	−0.65 (−1.0 to −0.3)	0.001	−0.49 (−0.9 to −0.1)	0.007	−0.38 (−0.7 to −0.01)	0.045
GCS, apical, %	−0.87 (−1.4 to −0.3)	0.003	−0.62 (−1.2 to −0.1)	0.028	−0.16 (−0.7 to 0.4)	0.57
Torsion, degrees	0.44 (0.08 to 0.80)	0.018	0.38 (0.03 to 0.7)	0.034	0.28 (−0.08 to 0.64)	0.13

CMR indicates cardiac magnetic resonance imaging; GCS, global circumferential strain; LAEF, left atrial emptying fraction; LA_max_, left atrial maximal volume; LA_min_, left atrial minimal volume; LV, left ventricular; LVEDV, left ventricular end-diastolic volume; LVEF, left ventricular ejection fraction; LVESV, left ventricular end-systolic volume; LVM, left ventricular mass; LVSV, left ventricular stroke volume; RAEF, right atrial emptying fraction; RAmax, right atrial maximal volume; RAmin, right atrial minimal volume; RVEDV, right ventricular end-diastolic volume; RVEF, right ventricular ejection fraction; RVESV, right ventricular end-systolic volume; and RVSV, right ventricular stroke volume.

* Multivariable model: age, sex, systolic blood pressure, diastolic blood pressure, physical activity, smoking, alcohol, body mass index, use of blood pressure medication, use of cholesterol medication, and ethnicity.

As shown, there was no difference in LVEF between participants with and without DM. Following full multivariable adjustments, however, DM was associated with smaller LVEDV_Indexed_ and smaller LVSV_Indexed_. Although there was no difference in LVM_Indexed_, mass/volume ratio was significantly greater in DM versus no DM participants.

LV GCS and torsion measures are displayed in Tables 2 and 3. In age- and sex-adjusted models, both mid GCS (GCS_Mid) and apical GCS (GCS_Apex) were lower in DM. Following multivariable adjustments, GCS_Mid remained associated with impaired strain in DM, while basal and apical strain measures were not significantly different between participants with and without DM. For LV torsion, unadjusted and age- and sex-adjusted models showed increased torsion in participants with DM. Following multivariable adjustments, however, the difference no longer reached statistical significance.

### LA Morphology and Function—DM versus No DM

In the age- and sex-adjusted models, there were no significant differences in LA measures. Following full multivariable adjustments, DM was associated with smaller LAmax_Indexed_. LAmin_Indexed_ was not related to DM status. Left atrial emptying fraction (LAEF) was lower in DM compared to participants with no DM.

### Right Ventricular Morphology and Function—DM Versus No DM

Similar to LV measures, RVEF did not differ between participants with and without DM. However, as with LV dimensions, RVEDV_Indexed_ and RVSV_Indexed_ were smaller in the DM population.

### RA Morphology and Function—DM Versus No DM

In both age- and sex-adjusted models, and following multivariable adjustments, RA measures were all significantly different in DM versus no DM. Thus, both RAmax_Indexed_ and RAmin_Indexed_ were smaller in participants with DM. In addition, right atrial emptying fraction was significantly lower in participants with DM.

### Sensitivity Analysis

In a sensitivity analysis, we performed propensity score matching using covariates from the multivariable model to match participants without DM to participants with DM. The treatment effects from the propensity score matching were essentially similar to the linear regression coefficients. Thus, for the LVEDVI the effect size was estimated to (average treatment effect on the treated [95% CI]) −2.61 (−6.1 to 0.8), *P*=0.14, n=3654; and for LAEF, the effect size was estimated to −7.85 (−12.9 to −2.8), *P*=0.002, n=3680.

## Discussion

In the present study, individuals without known CVD and with preserved LVEF from the UK Biobank, a low-risk general population study, were examined to study early changes in cardiac morphology and function associated with DM. The findings of this comprehensive study suggest that DM is associated with discrete but significant cardiac remodeling affecting all 4 cardiac chambers. Thus, in contrast to the current paradigm,^35^ our findings suggest that diabetic cardiomyopathy is not a regional condition of the LV but affects the heart globally. These changes can be observed before impairment in LVEF or RVEF occurs and before manifest heart disease develops. The present findings, therefore, significantly add to our current understanding of early cardiac alterations related to DM and open a new direction for early detection and research into diabetic cardiac complications.

CVD is the most common complication in DM, which is the reason why both American and European DM and cardiology associations have developed common recommendations for detecting, preventing, and treating CVD in DM. Present guidelines, and the majority of research, have so far described diabetic cardiomyopathy as a disease typically related to the LV.^35^

In 1972, Rubler et al^6^ presented evidence of a special myocardial involvement in DM from autopsies of 4 patients with heart failure, DM, and kidney disease without major disease of the coronary arteries. Research in the last decades have demonstrated that early changes can be detected in the diabetic LV using conventional echocardiography, tissue Doppler imaging, and deformation imaging.^9–11^

In the present study of changes in LV morphology and function, findings from previous decades of research are confirmed in that we find subtle changes in LV volumes and in the relationship between LV mass-to-volume. These findings are important as they demonstrate that subtle changes are present even before LV mass increases and before impairment in LVEF, and these changes are associated with adverse events.^36^ Furthermore, using CMR-tagging, which is considered the gold standard for CMR deformation imaging,^37^ we find global strain to be impaired even in the presence of normal LVEF. Thus, these findings correspond to studies of type 2 DM,^16^ heart failure with preserved ejection fraction patients,^38^ and findings from type 1 DM without known heart disease^11^ studied with speckle-tracking echocardiography, and other similar research.

Changes in the RV associated with DM remain largely unexplored; the present study, therefore, provides a novel direction for future research. Parallel to our findings in the LV, we find that, while RVEF was unaffected, there were significant changes in RV volumes, which were smaller in individuals with DM. Recent reports in smaller populations have suggested changes in RV related to DM: Patscheider et al^18^ found smaller RV volumes using CMR but only in men and not in women, and Widya et al^39^ found similar findings but only studied men. In contrast, we find RV morphology to be altered similarly in both men and women with DM.

In the present study, we find smaller LA volumes and lower LAEF associated with DM. Low LAEF has previously been shown to be a strong predictor of atrial fibrillation, which is common in patients with DM.^40,41^ With the current understanding of diabetic cardiomyopathy, the finding of smaller LA volumes seems counterintuitive in that larger atrial volumes would be expected in relation to possibly increased LV filling pressures. It is important, however, to remember that the present findings represent very early cardiac changes associated with DM. In The Thousand & 1 Study, a population of 1100 type 1 DM patients without known heart disease with a mean DM duration of 25 years, LA volume indexed for body surface area was not different when compared to 200 healthy controls.^11^ LA enlargement, therefore, probably represents a later finding in the pathogenesis of diabetic cardiomyopathy. Significantly, the present finding of lower LAEF indicates that reduction in LA function develops before impairment in LV function. In addition, RA morphology and function were examined in the present study. Here, RA volumes were found to be smaller in DM, and right atrial emptying fraction was found to be lower. To the best of our knowledge, this study is the first to systematically report alterations in RA morphology and function in relation to DM.

In summary, CVD is the most important complication in DM and early detection of cardiac involvement is of pivotal importance. In the present study of 3984 participants without known heart disease and with preserved LVEF from the UK Biobank Cardiac Magnetic Resonance Substudy, early alterations in cardiac morphology and function were observed. Specifically, DM was associated with alterations in all 4 cardiac chambers. LV volumes were smaller and mass-to-volume ratios were larger in DM before differences in LVEF or LV mass could be detected. Furthermore, subtle changes in cardiac LV deformation could be detected using CMR-tagging even in the presence of preserved ejection fraction. DM was also associated with smaller right ventricular volumes without changes in right ventricular ejection fraction. A consistent pattern also emerged for both atria, demonstrating smaller LA volumes, smaller RA volumes, and poorer function with lower emptying fractions. The present findings, therefore, corroborate previous findings of changes in the LV and extend current knowledge of diabetic alterations to include changes in the RV, LA, and RA.

Pathophysiological considerations should briefly be considered. A possible contributing mechanism for the smaller chamber sizes could be increased fibrosis and ventricular hypertrophy, and thereby relative enlargement in wall thickness.^42^ Also, DM is associated with cardiac autonomic neuropathy, increased blood pressure, and metabolic disturbances, leading to increased resting heart rate,^43^ as seen in the present study. It is possible that increases in resting heart rate contribute to an initial remodeling and thereby smaller chamber sizes to match cardiac output with circulatory requirements. A similar mechanism could be at play in pulmonary disease where smaller chamber sizes^44,45^ are matched by increases in resting heart rate.^46^ It is, however, also possible that smaller chamber sizes contribute to higher resting heart rates as causality cannot be determined from the present study. Furthermore, there may be a mechanical explanation in which chambers sizes are decreased to maintain ejection/emptying fraction.^47^ Other unexplored mechanisms may also be at play and should be studied in future research.

Possible limitations should be considered. First, the UK Biobank population represents a low-risk cohort and have been shown to be healthier than the background population, as demonstrated by the low prevalence of DM compared with data from the Health Survey for England.^48^ The findings, therefore, possibly represent changes earlier in the natural history of diabetic cardiomyopathy compared to other populations. Second, the population consisted mainly of ethnically white (96%). Although interaction analyses did not reveal a difference in the relationship between cardiac changes, DM, and ethnicity, findings may be different in other populations. Third, there could have been a misclassification of participants with unknown or unreported DM as not having DM. This, however, would draw the findings toward to null-hypothesis and can, therefore, not explain our findings. Last, there may be subclinical ischemic heart disease, which we cannot account for.

In conclusion, diabetic cardiomyopathy is a global condition affecting all 4 chambers of the heart. In the early phase, DM is associated with smaller cardiac chambers as well as discrete or subclinical impairments in chamber functions. These findings represent a shift in the understanding of diabetic cardiomyopathy, and, if confirmed in other studies, are a significant step forward in identifying early myocardial changes related to DM.
